# Resilience and professional identity among young healthcare workers in a Shanghai megahospital during COVID-19: a cross-sectional study

**DOI:** 10.3389/fpubh.2025.1632053

**Published:** 2025-07-17

**Authors:** Di Xu, Yaxu Zhou, Yuru Yan, Yan Wang, Ye Le, Yuchun Ma, Chunming Wang, Ying Zhou

**Affiliations:** ^1^Nursing Department, Renji Hospital, School of Medicine, Shanghai Jiao Tong University, Shanghai, China; ^2^Finance Department, Renji Hospital, School of Medicine, Shanghai Jiao Tong University, Shanghai, China; ^3^Smart Hospital Development Department, Renji Hospital, School of Medicine, Shanghai Jiao Tong University, Shanghai, China

**Keywords:** professional identity, young healthcare workers, humanistic care, working conditions, organizational resources, public health emergency, COVID-19

## Abstract

**Background:**

Public health crises such as the COVID-19 pandemic place immense psychological and professional pressure on healthcare workers, particularly those early in their careers. While much research has focused on burnout and stress, fewer studies have examined the role of professional identity and its organizational determinants among actively employed young healthcare professionals.

**Methods:**

This cross-sectional study surveyed 326 young healthcare workers (≤40 years old) across clinical, research, and administrative roles in a megahospital in Shanghai, China. A validated multidimensional scale was used to assess professional identity. Independent variables included job satisfaction, workload, exposure to high-risk duties, and perceived organizational support (including satisfaction with humanistic care and working conditions). Descriptive statistics, Mann–Whitney U and Kruskal-Wallis H tests, and multivariate linear regression were employed.

**Results:**

The majority of respondents (92.9%) reported a high level of professional identity (median score: 81, IQR: 13). Satisfaction with humanistic care (*B* = 3.431, *p* = 0.009) and working conditions (*B* = 3.679, *p* = 0.003) were strong predictors of higher professional identity scores. Significant differences in professional identity were observed based on gender, income satisfaction, and exposure to COVID-19 patients.

**Conclusion:**

This study highlights the essential role of organizational resources—particularly perceived humanistic care and adequate working conditions—in sustaining professional identity among young healthcare professionals during public health emergencies. The findings underscore the need for system-level interventions that support resilience and workforce stability. These insights may inform global strategies for healthcare human resource management in high-pressure contexts.

## Background

The professional identity of young medical practitioners in major hospitals is a pivotal component of healthcare systems, particularly during public health crises. Grasping the elements that mold this identity is vital for crafting robust support mechanisms and policies that safeguard the well-being and career growth of emerging medical professionals. Public health crises, exemplified by the COVID-19 pandemic, underscore the vulnerabilities and trials faced by healthcare personnel, underscoring the necessity of exploring their professional identity under such conditions.

Public health emergencies profoundly affect the professional identity of healthcare workers, touching upon various facets of their professional and personal realms. These workers contend with heightened risks of stress, burnout, moral injury, depression, trauma, and other psychological adversities during such crises (1). The psychological state of healthcare personnel deteriorates due to job insecurity, extended isolation, and uncertainty about the future, particularly affecting younger employees and those with advanced educational backgrounds (2). Many healthcare workers maintain a strong commitment to their duties despite personal risks, a sentiment that holds irrespective of professional status (3). These dynamics collectively forge the professional and personal landscapes of healthcare workers during emergencies, like COVID-19.

Recent strides in understanding the occupational identity in healthcare are comprehensive, spanning dimensions such as identity formation, the effects of interprofessional education, and the impact of workplace environments. The formation of a professional identity is critical for the well-being of healthcare professionals and the caliber of care they deliver (4). The integration of interprofessional education into healthcare training has been recognized as a significant element in shaping professional identities, fostering cooperation and mutual understanding across different medical fields (5). Moreover, the work environment plays a vital role in nurturing and sustaining professional identity, with factors like workload, resource availability, and organizational culture influencing healthcare workers’ perceptions of their roles (6). Young medical professionals encounter a spectrum of challenges during public health crises, broadly classified into practical, psychological, and educational obstacles. Practical challenges encompass the correct utilization of personal protective equipment (PPE) and assumptions regarding the availability of medical students for volunteer efforts during emergencies (7, 8). Psychological challenges include an increased risk of burnout and moral injury, with a significant impact on mental health due to stressful conditions (7, 9). Educational challenges involve the disruption of clinically-based medical education and the necessity for disaster training programs to enhance preparedness (10–12).

Support from hospital administration is crucial in shaping the occupational identity of healthcare workers. This support influences various aspects of their professional lives, enhancing job satisfaction, mental well-being, and the capacity to manage occupational stress. A sense of belonging to their hospital, beliefs in their organization’s efficacy, and effective communication with patients are positively associated with job satisfaction and mental well-being (13). The dual identity model suggests that interprofessional collaboration between nurses and physicians could improve if these professionals identify with both their professional category and care unit (14). Support from hospital administration can also mitigate the development of adversarial unprofessional identities and foster a more collaborative work environment (15). In addressing these challenges, our study investigates the current state and influencing factors of the occupational identity of medical youth in large general hospitals in Shanghai amidst public health emergencies. Employing a mixed-methods design, this study integrates quantitative survey data with thematic insights to examine how professional identity develops among young healthcare professionals during public health emergencies. Key dimensions of interest include job satisfaction, perceived stress, organizational support, and individual resilience.

To interpret these complex interactions, we adopt the Job Demands–Resources (JD-R) model as the theoretical framework. The JD-R model explains how job demands (e.g., workload, emotional strain) and job resources (e.g., humanistic care, administrative support) interact to shape employee well-being, motivation, and engagement. By applying this model in the context of the COVID-19 crisis, our study moves beyond surface-level description to explore the structural and psychological mechanisms by which professional identity is either strengthened or strained. Our findings elucidate the significant impact of workload, perceived support, resilience, and motivation on the occupational identity of young medical professionals. High levels of workload are consistently linked with increased stress, negatively affecting job satisfaction and overall well-being. Perceived support from hospital administration proves critical in maintaining job satisfaction and mitigating stress. Personal attributes such as resilience and motivation also play crucial roles in shaping occupational identity, with resilient and motivated individuals more likely to exhibit a robust professional identity (16). In addition to focusing on professional identity as the primary outcome, this study also draws conceptually on the notion of resilience. In the context of the JD-R model, resilience is understood not as a measured variable but as a psychological capacity that enables individuals to cope with high job demands when supported by adequate resources. This framework helps interpret how organizational support and humanistic care may indirectly sustain or strengthen professional identity, especially under the pressures of public health emergencies.

This research delineates the journey from data collection to analysis, highlighting the essential role of support systems in enhancing the occupational identity of young medical professionals during public health crises. Our results underscore the need for effective support systems and interventions to sustain the well-being of healthcare workers during emergencies. By understanding the factors influencing occupational identity, healthcare systems can implement strategies to boost job satisfaction, reduce stress, and enhance overall well-being among medical professionals amid public health crises.

## Methods

### Demographic characteristics survey

A structured questionnaire was designed to collect demographic data from a broad spectrum of medical professionals at a large general hospital in Shanghai. This included doctors, nurses, technical staff, research personnel, and management staff. Utilizing convenience sampling, 380 young medical workers were selected for the survey during June and July 2022. After excluding incomplete or erroneous questionnaires, a total of 326 valid responses were collected, resulting in an effective response rate of 85.8%. The criteria for inclusion of young medical workers in the study were: (1) age ≤40 years; (2) no history of organic diseases, mental illnesses, or psychological disorders; and (3) voluntary participation in the study. The survey was conducted online using digital questionnaire tools Sojump. Data collection spanned 4 weeks, with all responses being anonymized to maintain confidentiality. This study involving human participants was reviewed and approved by the Ethics Committee of Renji Hospital, Shanghai Jiao Tong University School of Medicine (EX-2025-011). All procedures performed in this study involving human participants were in accordance with the ethical standards of the institutional research committee and with the 1964 Helsinki Declaration and its later amendments. Written informed consent was obtained from all participants prior to data collection. The participants were informed about the voluntary nature of their participation, the purpose of the study, data confidentiality, and their right to withdraw at any time. The survey was conducted anonymously through a secure online platform (Sojump), and all data were de-identified to ensure privacy and confidentiality.

### Questionnaire design

Based on a literature review and group discussions, a self-designed structured questionnaire was created, divided into three parts: ① Basic information of the respondents, including gender, age, marital status, and political affiliation. ② Impact of Emergency Public Health Events: This section aimed to assess the respondents’ work-related experiences under the influence of public health emergencies. Items included whether they had contact with suspected or confirmed patients, whether they underwent medical observation or closed-loop management, and their involvement in epidemic prevention and control work. ③ Professional identity: The professional identity scale was based on the scale (17) and was modified by us. The scale consists of 21 items across six dimensions: professional cognition (4 items), professional emotion (3 items), professional expectations (3 items), professional commitment (4 items), professional behavior (4 items), and professional values (3 items). Each item uses a 5-point Likert scale, ranging from “completely disagree” to “strongly agree,” corresponding to scores from 1 to 5. The sum of the item scores gives the total professional identity score, ranging from 21 to 105. A total score ≥ 63 (60% of the maximum score) indicates a high level of professional identity, while a total score < 63 indicates a low level. The Cronbach’s *α* coefficient for the scale was 0.913, with a KMO value of 0.898 and a Bartlett’s test result of *χ*^2^ = 4290.60, *p* < 0.01, indicating high reliability and validity of the scale. The Cronbach’s α values for the six dimensions ranged from 0.804 to 0.881.

In this study, the selection and operationalization of variables were informed by the Job Demands–Resources (JD-R) model. Specifically, job demands were assessed through indicators such as perceived workload and direct exposure to COVID-19 patients. Job resources included satisfaction with working conditions, satisfaction with humanistic care, and perceived support from hospital administration. Perceived support from hospital administration, measured via the item “I feel that the hospital provides adequate administrative support during public health emergencies,” was used to represent the organizational support dimension in the JD-R framework. To ensure consistency with the Job Demands–Resources (JD-R) theoretical framework, the questionnaire variables were conceptually mapped into two core categories. Job Demands included (1) extended working hours and (2) direct contact with suspected or confirmed COVID-19 patients. Job Resources included (1) satisfaction with the hospital’s humanistic care, (2) satisfaction with working conditions during public health emergencies, (3) perceived adequacy of institutional support, and (4) monthly income level. These variables were selected to reflect organizational and psychological environments influencing individual outcomes. The professional identity score was treated as a reflective indicator of job outcomes in the JD-R model.

### Departmental comparison of occupational identity

To examine variations in occupational identity across different demographic and work-related subgroups, the respondents were stratified based on characteristics such as gender, education level, professional title, income satisfaction, contact with confirmed or suspected COVID-19 cases, and perceived support from hospital administration. The median occupational identity scores and interquartile ranges (M [IQR]) were calculated for each subgroup. Differences in scores were analyzed using Mann–Whitney U tests for two-category variables and Kruskal-Wallis H tests for variables with more than two categories. This analysis aimed to identify significant demographic and contextual factors influencing professional identity among young healthcare workers.

### Impact of emergency public health events

The impact of emergency public health events on job satisfaction, workload, and stress levels was assessed through survey questions. Participants were asked to rate the impact of recent public health emergencies, such as the COVID-19 pandemic, on their job satisfaction, workload, and stress levels. These questions were designed to capture changes in these variables before, during, and after the emergency events. The responses were analyzed to identify trends and correlations between the impact of public health emergencies and occupational identity.

### Perceived support from hospital administration

Perceived support from hospital administration was measured using a series of survey questions that assessed participants’ perceptions of mental health support, communication, resource availability, and preparedness training provided by their hospital administration. Participants rated their agreement with statements related to these aspects on a Likert scale. The responses were analyzed to determine the level of perceived support and its correlation with occupational identity. The data were used to identify areas where hospital administration could improve support for medical professionals during public health emergencies.

### Statistical analysis

The statistical data analysis was carried out using SPSS Statistics 25 (IBM, Armonk, NY) software. The data are presented as median and interquartile range (M [IQR]). Frequency and composition ratios were used to describe the basic information of the respondents. The Shapiro–Wilk test showed that the total score of professional identity and the scores of each dimension were non-normally distributed (W values between 0.864 and 0.983, *p* < 0.01). Therefore, median (M) and interquartile range (IQR) were used to describe the professional identity scores. Mann–Whitney U tests (for comparisons between two groups) and Kruskal-Wallis H tests (for comparisons among multiple groups) were used to compare differences in total professional identity scores between subgroups. Based on the results of Mann–Whitney U tests and Kruskal-Wallis H tests, variables with a significance level of *p* < 0.1 were directly selected and entered into the multiple linear regression model, with the total professional identity score as the dependent variable. Each survey response was considered as an individual data point. All statistical analyses used two-sided tests with *p* < 0.05 was considered statistically significant.

## Results

### Demographic characteristics

**Figure 1:**
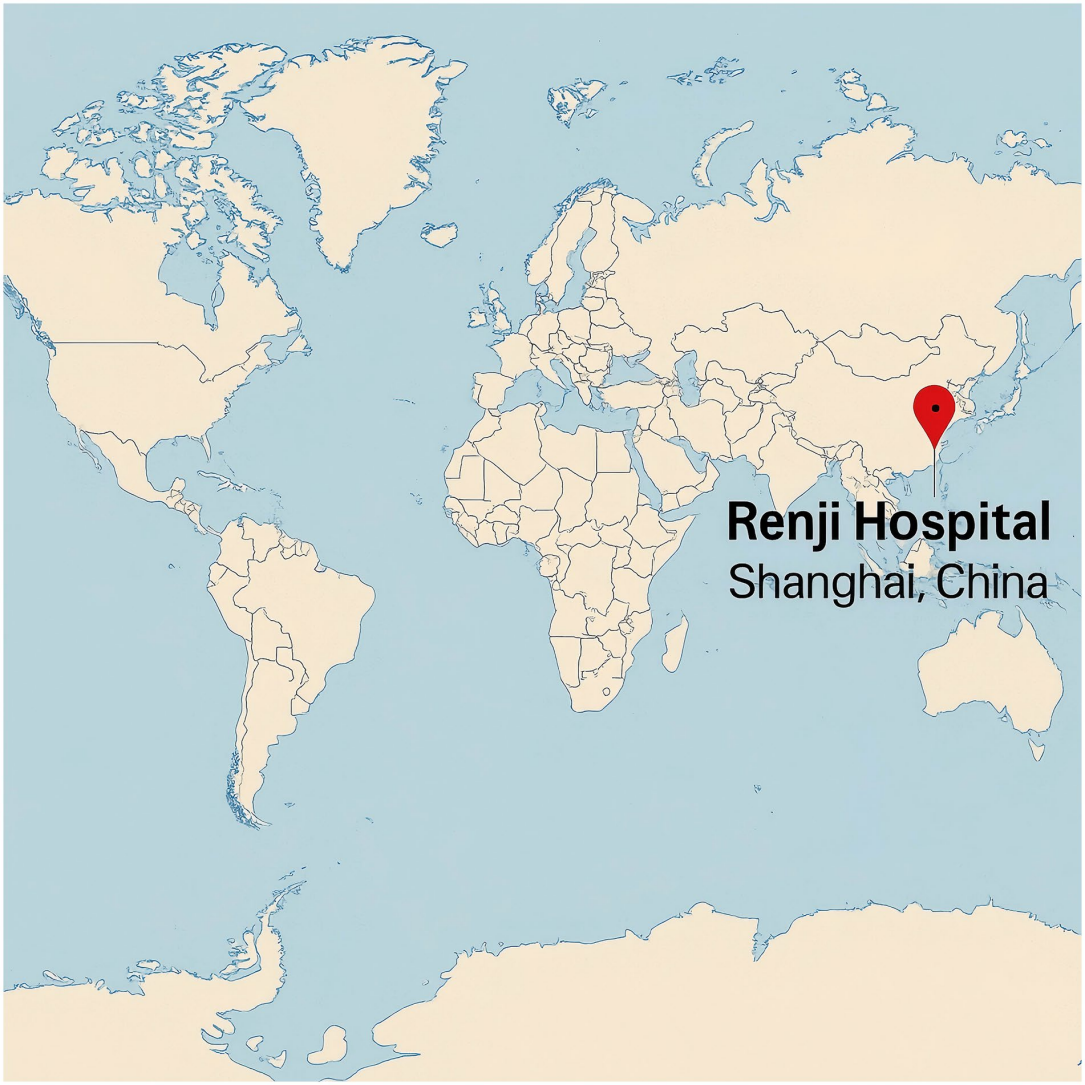
Geographic location of Renji hospital. Renji hospital is located in Pudong New Area, Shanghai, China. The map indicates the precise location of the hospital, highlighting its position within Shanghai to contextualize the study setting.

A total of 326 young healthcare workers were surveyed, of whom 32.8% were doctors and 26.4% were nurses. The majority were female, aged between 26 and 35 years, married, and had 1–5 years of work experience. Most respondents held a bachelor’s degree or higher and a junior professional title. Approximately 48.2% had a monthly income exceeding 10,000 RMB, although 64.4% expressed dissatisfaction with their income. The majority (68.1%) reported working 40–60 h per week. The geographic location of Renji Hospital is shown in Figure 1, and further details on participant characteristics are summarized in Table 1.

**Table 1:** Demographic and sociological characteristics.

Variable	Number of people	Composition ratio/%
Gender		
Male	109	33.40
Female	217	66.60
Age		
Under 25 years	55	16.87
26–30 years	103	31.60
31–35 years	98	30.06
36–40 years	70	21.47
Marital status		
Married	180	55.21
Unmarried	146	44.79
Political affiliation		
Communist party member	109	33.44
Communist youth league member	79	24.23
Masses	138	42.33
Work experience		
1–5 years	159	48.77
6–10 years	78	23.93
11–15 years	53	16.26
Over 16 years	36	11.04
Occupation		
Doctor	107	32.82
Nurse	86	26.38
Professional Technical Personnel	58	17.79
Research	45	13.80
Management	30	9.20
Education level		
Associate degree	42	12.88
Bachelor’s degree	112	34.36
Master’s degree	90	27.61
Doctoral degree	82	25.15
Title		
Non-graded	96	29.45
Junior	150	46.01
Intermediate	70	21.47
Senior	10	3.07
Monthly income		
Below 4,999 yuan	52	15.95
5,000–7,999 yuan	52	15.95
8,000–9,999 yuan	65	19.94
≥10,000 yuan	157	48.16
Satisfaction with income		
Yes	116	35.58
No	210	64.42
Work duration		
<40 h/week	37	11.35
40-60 h/week	222	68.10
>60 h/week	67	20.55

### Impact of demographic and sociological characteristics

The overall professional identity score among young healthcare workers was 81 (IQR = 13), with an average of 3.77 (SD = 0.52) across all dimensions. The highest mean scores were observed in the professional behavior (*M* = 4.26, SD = 0.51) and professional values (*M* = 4.04, SD = 0.73) dimensions, while professional cognition scored relatively lower (*M* = 3.26, SD = 0.79), suggesting greater internalized commitment than perceived external recognition. Notably, 92.9% of respondents (303/326) reported high levels of professional identity (total score ≥ 63).

To explore subgroup variation, we compared identity scores across key demographic and occupational factors (Table 2). Significant differences were observed for gender (*p* = 0.021) and income satisfaction (*p* = 0.001), with male respondents and those satisfied with their income reporting higher scores. Although not statistically significant, respondents with <40 working hours or those in technical staff roles also exhibited higher identity scores, hinting at possible trends.

**Table 2:** Comparison of total professional identity scores across demographic groups.

Variable	M (IQR)	Z/H	*p*
Gender		−2.310	0.021
Male	83 (13)		
Female	80 (12)		
Age		2.914	0.405
Under 25 years	79 (13)		
26–30 years	82 (15)		
31–35 years	81 (14)		
36–40 years	82 (13)		
Marital Status		−1.834	0.067
Married	82 (13)		
Unmarried	79 (13)		
Political affiliation		0.997	0.608
Communist party member	80 (14)		
Communist youth league member	81 (12)		
Masses	82 (13)		
Work Experience		2.784	0.426
1–5 years	81 (10)		
6–10 years	78 (17)		
11–15 years	82 (12)		
Over 16 years	82 (14)		
Occupation		8.768	0.067
Doctor	79 (12)		
Nurse	81 (15)		
Professional technical personnel	84 (16)		
Research	82 (11)		
Management	81 (10)		
Education level		1.262	0.738
Associate degree	82 (11)		
Bachelor’s degree	82 (14)		
Master’s degree	79 (12)		
Doctoral degree	81 (14)		
Title			
Non-graded	80 (13)	6.103	0.107
Junior	82 (14)		
Intermediate	81 (14)		
Senior	85 (7)		
Monthly Income		7.231	0.065
Below 4,999 yuan	77 (13)		
5,000–7,999 yuan	78 (17)		
8,000–9,999 yuan	81 (10)		
≥10,000 yuan	82 (13)		
Satisfaction with income		−3.445	0.001
Yes	82 (9)		
No	78 (15)		
Work duration		5.140	0.077
<40 h/week	83 (8)		
40-60 h/week	81 (13)		
>60 h/week	77 (17)		

**Figure 2:**
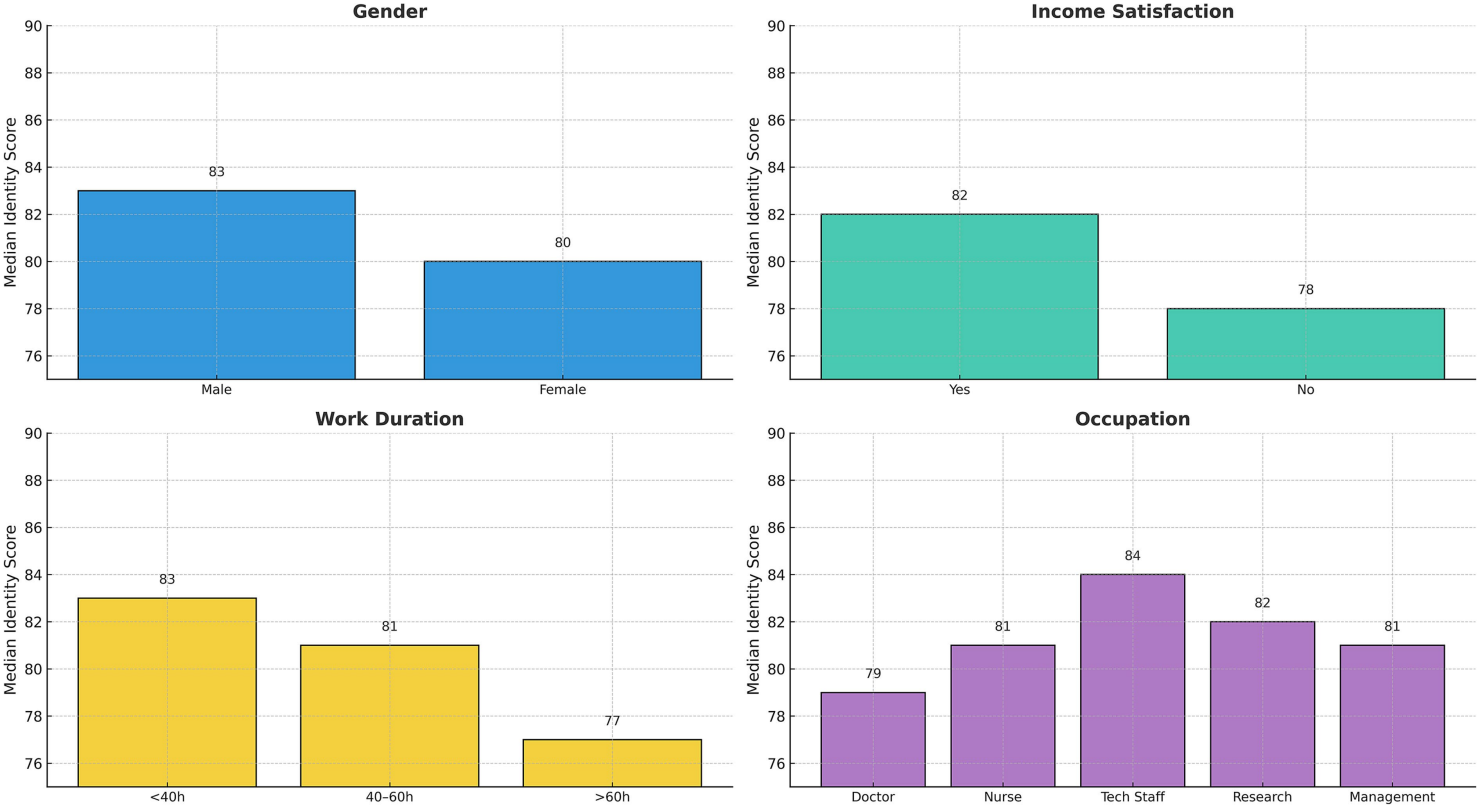
Median professional identity scores across key subgroups. Bar plots showing the median professional identity scores stratified by gender, income satisfaction, weekly working hours, and occupational type. Each bar represents the group median, with scores ranging from 77 to 84.

These distributions are visualized in Figure 2, which shows the median identity scores stratified by gender, income satisfaction, weekly work hours, and occupational type. Male participants and technical professionals scored highest (83 and 84, respectively), while the lowest scores were seen among those dissatisfied with income or working >60 h per week (both 77). These results highlight that income satisfaction and gender are significant contextual factors associated with professional identity among young healthcare workers, suggesting that economic perceptions and demographic characteristics may influence identity formation in this population.

### Impact of public health emergencies

As shown in Table 3, 44.20% of young healthcare workers had contact with suspected or confirmed COVID-19 patients, and 58.90% experienced medical observation or closed-loop management. A total of 39.30% of young healthcare workers participated in epidemic prevention and control efforts within their departments, with 14.70 and 16.00% being transferred to high-risk departments such as emergency/fever clinics or to designated hospitals/temporary hospitals (e.g., Fangcang hospitals) to support epidemic control efforts. Additionally, 42.00% of young healthcare workers worked continuously for more than 30 days during the public health emergency. A total of 69.30% reported that their work pressure significantly increased during the public health emergency. Meanwhile, 50.30 and 43.90% of young healthcare workers expressed satisfaction with the humanistic care and working conditions provided by their hospitals during the public health emergency, respectively. The results of the univariate analysis showed that the total professional identity score of young healthcare workers differed significantly in terms of contact with suspected or confirmed patients, and satisfaction with the humanistic care and working conditions provided by the hospital during the public health emergency (*p* < 0.05), while no statistically significant differences were found in other.

**Table 3:** Comparison of occupational identity scores among survey respondents with different work situations under the influence of public health emergencies.

Variable	Number of people	Composition ratio/%	M (IQR)	*Z*/*H*	*p*
Contact with suspected or confirmed patients				−2.145	0.032
Yes	144	44.17	80 (12)		
No	182	55.83	82 (14)		
Medical observation or closed-loop management				−0.128	0.898
Yes	192	58.90	81 (11)		
No	134	41.10	82 (16)		
Epidemic prevention and control work participation				1.143	0.887
Not participated	61	18.71	81 (12)		
Participated in volunteer work arranged by the unit	37	11.35	81 (13)		
Participated in the department	128	39.26	82 (13)		
Transferred to high-risk departments like emergency/fever clinic	48	14.72	80 (14)		
Transferred to designated hospitals/cabins and other units	52	15.95	81 (15)		
Continuous workdays during public health emergencies				3.955	0.412
5 Days	68	20.86	82 (11)		
6–10 Days	54	16.56	78 (16)		
11–20 Days	33	10.12	81 (10)		
21–30 Days	34	10.43	82 (14)		
>30 Days	137	42.02	82 (14)		
Work pressure during public health emergencies				0.942	0.624
Significantly increased	226	69.33	81 (14)		
Same as usual	84	25.77	82 (12)		
Significantly decreased	16	4.91	79 (13)		
Satisfaction with hospital’s humanitarian care during public health emergencies				46.055	0.000
Dissatisfied	29	8.90	69 (17)		
Neutral	133	40.80	77 (15)		
Satisfied	164	50.31	83 (12)		
Satisfaction with hospital’s working conditions during public health emergencies				46.502	0.000
Dissatisfied	43	13.19	69 (13)		
Neutral	140	42.94	79 (12)		
Satisfied	143	43.87	83 (12)		

### Regression analysis of influencing factors

**Figure 3:**
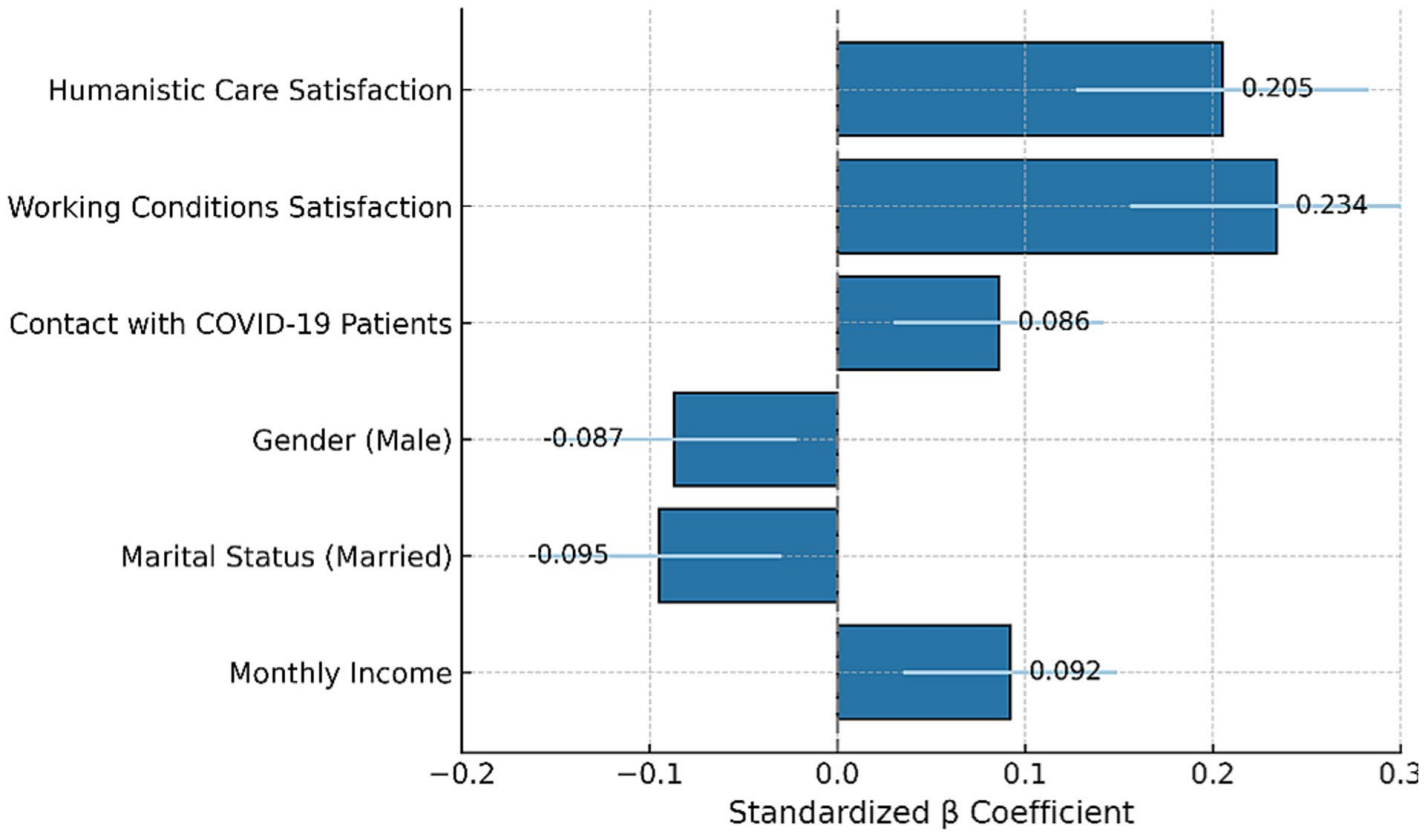
Predictors of professional identity. Standardized regression coefficients (*β*) with 95% confidence intervals from a multivariate linear regression model predicting total professional identity scores. The strongest predictors were satisfaction with working conditions and perceived humanistic care. Contact with COVID-19 patients, gender, marital status, and monthly income showed smaller effects. Error bars indicate ±1 standard error of *β*.

Using sociodemographic characteristics and work conditions as independent variables and the total professional identity score as the dependent variable. Based on the analysis results of the Mann Whitney U test and Kruskal Wallis H test mentioned above, 9 variables were selected as independent variables with *p* < 0.1 as the standard: gender, marital status, occupation, monthly income, income satisfaction, working hours, contact with suspected or confirmed patients, satisfaction with the humanistic care provided by the hospital during the public health emergency, and satisfaction with the working conditions provided by the hospital during the public health emergency. These variables were then included in a multiple linear regression analysis, with the total professional identity score as the dependent variable. Table 4 presents the variable assignments, and Table 5 shows the results of the multiple linear regression analysis. As shown in Figure 3, satisfaction with working conditions (*β* = 0.234, *p* = 0.003) and humanistic care (*β* = 0.205, *p* = 0.009) emerged as the most influential predictors of professional identity among young healthcare workers. By contrast, gender and marital status were negatively associated, albeit marginally, while monthly income and contact with suspected or confirmed patients showed a modest positive effect. These findings underscore the critical importance of organizational and relational factors in strengthening professional identity under crisis conditions.

**Table 4:** Variable assignments for the analysis of factors related to the occupational identity of young medical staff.

Variable	Assignment
Gender	1 = Male; 2 = Female
Age	1 = Under 25 years; 2 = 26–30 years; 3 = 31–35 years; 4 = 36–40 years
Marital status	1 = Married; 2 = Unmarried
Occupation	1 = Doctor;2 = Nurse;3 = Professional Technical Personnel;4 = Research;5 = Management
Monthly income	1 = Below 4,999 yuan; 2 = 5,000–7,999 yuan; 3 = 8,000–9,999 yuan; 4 = 10,000 yuan and above
Satisfaction with income	1 = Yes; 2 = No
Work duration	1 = Less than 40 h/week; 2 = 40–60 h/week; 3 = More than 60 h/week
Contact with suspected or confirmed patients	1 = Yes; 2 = No
Satisfaction with humanistic care provided by the hospital during public health emergencies	1 = Dissatisfied; 2 = Neutral; 3 = Satisfied
Satisfaction with working conditions provided by the hospital during public health emergencies	1 = Dissatisfied; 2 = Neutral; 3 = Satisfied

**Table 5:** Multiple linear regression results for factors related to the occupational identity of young medical staff.

Variable	*B*	S.E.	*β*	*t*-value	*p*-value
Constant	65.766	5.834		11.272	0.000
Gender	−2.008	1.152	−0.087	−1.743	0.082
Marital status	−2.076	1.169	−0.095	−1.776	0.077
Occupation	0.011	0.449	0.001	0.025	0.980
Monthly income	0.885	0.553	0.092	1.601	0.110
Satisfaction with income	−0.68	1.256	−0.03	−0.542	0.588
Working hours	−0.704	1.015	−0.036	−0.693	0.489
Contact with suspected or confirmed patients	1.868	1.174	0.086	1.591	0.113
Satisfaction with hospital’s humanitarian care during public health emergencies	3.431	1.304	0.205	2.632	0.009
Satisfaction with hospital’s working conditions during public health emergencies	3.679	1.247	0.234	2.95	0.003

*R* = 0.478, *R*^2^ = 0.229.

Notably, among all predictors analyzed in the multivariate model, satisfaction with hospital-provided humanistic care (*β* = 0.205, *p* = 0.009) and working conditions (*β* = 0.234, *p* = 0.003) emerged as the most significant factors influencing professional identity (Figure 3). These findings were not only statistically significant but also the strongest in magnitude among all standardized coefficients (Figure 3), suggesting that organizational and relational support played a more critical role than demographic or workload-related factors in shaping professional identity during the public health emergency.

### JD-R framework interpretation of findings

**Figure 4:**
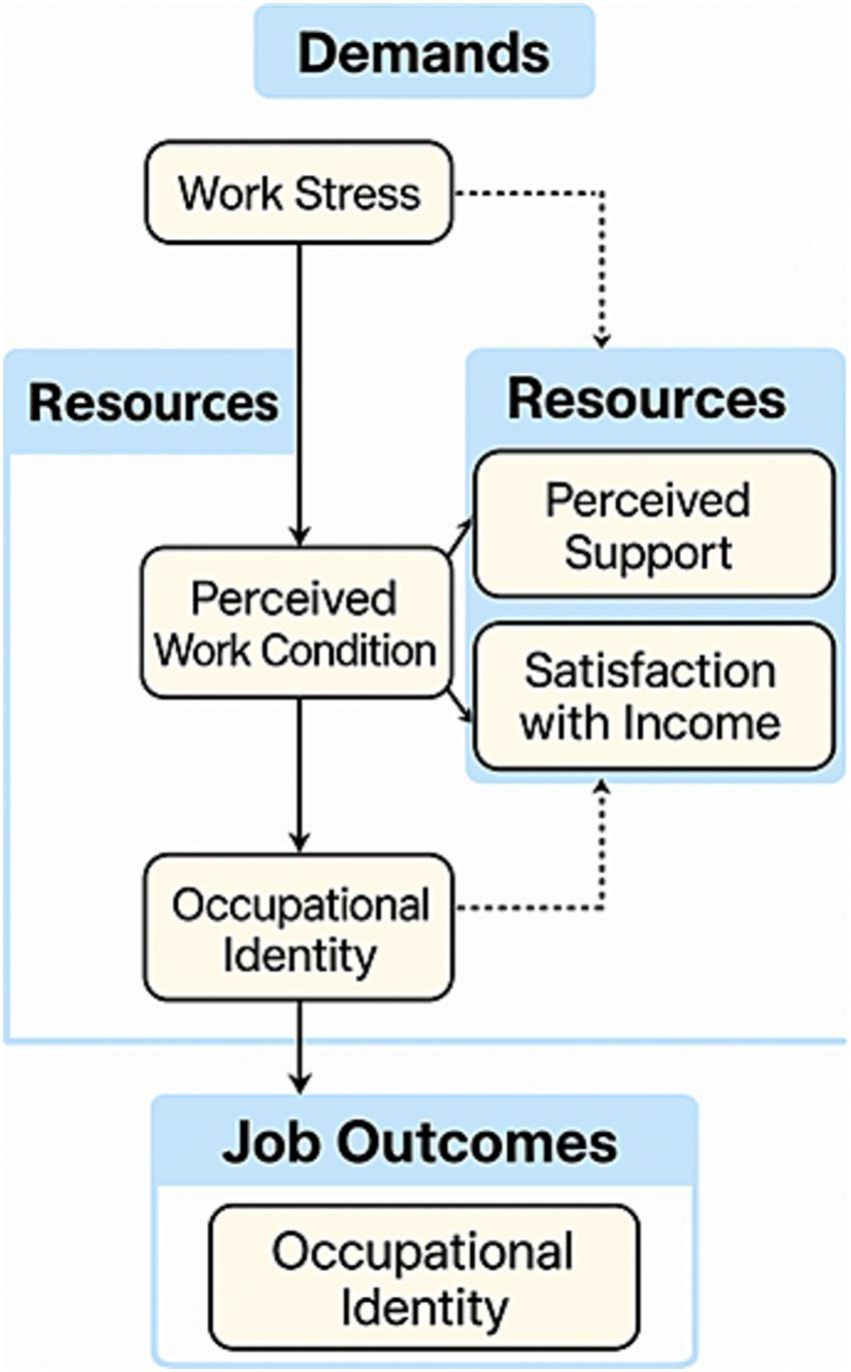
Conceptual JD-R pathway explaining professional identity formation. This figure illustrates a modified Job Demands–Resources (JD-R) model tailored to the context of young healthcare workers during public health emergencies. Solid lines represent statistically significant associations confirmed in this study (e.g., perceived work condition → occupational identity), while dashed lines indicate theoretically plausible but empirically non-significant relationships (e.g., work stress → occupational identity). Although the associations for job demands such as long working hours and direct exposure were not statistically significant, they are retained in the diagram to reflect their established theoretical relevance in JD-R literature. Occupational identity is shown both as a psychological construct shaped by resources and as an eventual job outcome, reflecting its dual role in professional development and engagement. Variables such as “organizational resources” in the diagram correspond to specific measured items in the manuscript, including satisfaction with humanistic care, adequacy of working conditions, and perceived support from hospital administration (measured by the item: “I feel that the hospital provides adequate administrative support during public health emergencies”). These terms were unified under theoretical constructs for conceptual clarity, consistent with the JD-R framework. Occupational identity appears twice to reflect its dual role—as both an intermediate psychological state influenced by work context and a final job-related outcome.

Our findings were interpreted through the lens of the Job Demands–Resources (JD-R) theoretical framework, which offers a useful model for understanding how workplace factors interact to shape professional outcomes. While job demands such as extended working hours and direct exposure to COVID-19 are widely recognized in prior literature as stress-inducing factors, our analysis did not find these variables to be statistically significant predictors of professional identity in this sample. Therefore, we refrained from asserting a direct negative impact of these job demands based on our data (Figure 4).

Conversely, two key organizational resources—satisfaction with humanistic care and perceived adequacy of working conditions during public health emergencies—demonstrated significant positive associations with professional identity. These findings are consistent with the JD-R model, which posits that organizational resources can foster employee motivation and psychological resilience. In this context, such resources may contribute to the development or reinforcement of professional identity among young healthcare workers. However, we acknowledge that the cross-sectional nature of our study limits causal interpretations, and the interaction between job demands and resources remains theoretical rather than empirically established in this study.

## Discussion

The application of the Job Demands–Resources (JD-R) model in this study provides a theoretically grounded and empirically supported framework for interpreting the occupational identity of young healthcare professionals during public health emergencies. As a flexible and widely adopted model in occupational psychology, the JD-R framework enables a comprehensive understanding of how job demands (e.g., prolonged working hours, exposure to COVID-19) interact with organizational resources (e.g., humanistic care, supportive working conditions) to influence employee well-being and performance. In the context of our study, this model is particularly relevant given the heightened psychosocial stressors faced by frontline medical staff and the variability of institutional responses during crises. Integrating JD-R not only contextualizes our findings within a well-validated theoretical structure but also underscores the need for system-level interventions that enhance workplace resources to buffer against professional fatigue and identity erosion. The JD-R lens thus allows us to move beyond individual-level explanations and emphasize modifiable organizational factors that sustain professional identity and resilience in early-career healthcare workers.

This study also contributes significantly to the existing body of knowledge on occupational identity and public health emergencies in China. In addition to describing the overall status of professional identity, our study identified two key organizational factors—satisfaction with humanistic care and satisfaction with working conditions during public health emergencies—as significant positive predictors of occupational identity among young healthcare workers. These findings align with the motivational process proposed by the Job Demands–Resources (JD-R) model, wherein the presence of adequate job resources enhances employees’ psychological engagement and identity consolidation, particularly in high-stress environments (18, 19). Humanistic care reflects the institution’s recognition of employees’ intrinsic needs, fostering a sense of being valued and respected, which contributes to the internalization of professional roles (20). While resilience was not directly measured in this study, our findings suggest that these key job resources—such as humanitarian care and institutional working conditions—may play a resilience-supportive role. Drawing on the JD-R framework, these resources likely help mitigate the stress induced by overwhelming job demands and promote adaptive coping strategies. This buffering and adaptive process, in turn, could indirectly reinforce professional identity by enabling workers to better withstand adversity and maintain a sense of purpose and competence. This perspective aligns with prior research suggesting that institutional support can buffer against burnout and identity erosion during crises. Likewise, satisfactory working conditions—especially under crisis—serve not only to reduce perceived stress but also to enhance a sense of professional control and meaning. These mechanisms may help explain how institutional support directly strengthens professional identity, especially when job demands are high but manageable. Conversely, factors like extended work hours and direct exposure to COVID-19 patients, although theoretically stress-inducing, did not show significant associations in our sample. Similar inconsistencies were observed in other international studies, where job demands varied in predictive strength across healthcare systems due to contextual differences in organizational buffers and cultural expectations (21). Furthermore, as noted by Luo & Mao (22), identity formation during emergencies may not directly follow the severity of workload, but instead be shaped by how these experiences are cognitively and socially interpreted. These results collectively underscore the importance of resource-based strategies—rather than solely workload mitigation—in sustaining professional identity and workforce retention among early-career healthcare professionals.

This study offers new insight into the formation and maintenance of professional identity among young healthcare workers during public health emergencies. While previous research has primarily focused on student populations or single professions, our findings reveal the heterogeneity of experiences across a broader group of young professionals actively employed in large general hospitals, including those in non-clinical roles. This broader perspective enables the identification of subtler but impactful influences—such as administrative transfers, high-risk duty assignments, and prolonged closed-loop management—that have been largely overlooked in earlier studies. Conducted in a megahospital in Shanghai, our study captures the current state and key determinants of professional identity during the COVID-19 pandemic, offering timely and context-rich evidence at a moment when global healthcare systems continue to face ongoing strain and uncertainty. By examining both individual and organizational factors, this research contributes to a more comprehensive understanding of how to support the resilience and retention of early-career healthcare workers in times of crisis.

In this study, the median total score for professional identity among young medical professionals was 81, with an average score of 3.77. A total of 92.9% of young medical professionals had a total score of ≥63, indicating a high level of professional identity under the influence of public health emergencies. Currently, there are numerous studies on the professional identity and influencing factors of specific personnel under public health emergencies, but most focus on medical students and nursing students who have not yet entered the workforce (23–25), with some researches targeting clinical nurses (26, 27). This study is novel in selecting young medical professionals across five different personnel sequences as the survey subjects. Another report used a similar professional identity scale to survey 289 clinical nurses during the COVID-19 pandemic, with an average score of 3.73 for the 30 items (28), which is close to the average score of young medical professionals in this study. This study also contributes significantly to the existing body of knowledge on occupational identity and public health emergencies in China (29, 30). It provides empirical evidence on the factors influencing occupational identity among medical youth, highlighting the important role of workload, perceived support, resilience, and motivation. These findings align with previous research that has documented the impact of these factors on job satisfaction and well-being among healthcare workers (31–35). For example, another study reached similar conclusions, noting that the total and dimensional scores of professional identities among clinical nurses during the COVID-19 pandemic were higher than the norms (32). Meanwhile, other researches also suggest that public health emergencies and natural disasters can positively impact the professional identity and ethics of healthcare workers (33–35).

Moreover, the study results show that young medical professionals scored differently across dimensions such as professional cognition, professional emotion, professional expectations, professional commitment, professional behavior, and professional values. The score for the professional cognition dimension was relatively low, with a total score of 13 (5) and an average of 3.26 (0.79). This dimension mainly evaluates the young medical professionals’ perception of the social status of healthcare workers, the value of their work, and the level of respect they receive. This result is similar to the findings of study found that standardized training nurses had a low evaluation of their professional cognition during the COVID-19 pandemic (36, 37). They pointed out that this might be because these nurses are relatively young, with shorter work experience, leading to insufficient and shallow professional cognition. The subjects in the study are young medical professionals, most of whom have 1–5 years of work experience and hold junior titles. Since the training period for healthcare professionals is long, they may not yet feel a strong sense of social status or value at the early stages of their careers. Therefore, efforts should be made to improve the career development channels for young medical professionals to enhance their social status. This study also found that the higher the satisfaction with income, the higher the professional identity. Income is also one aspect of social status, and improving hospital performance distribution, ensuring that excellent, reliable young medical professionals are rewarded fairly, and increasing their income can enhance their social status and improve their evaluation of professional cognition, thus boosting their professional identity. Additionally, establishing a youth talent development fund, offering opportunities for training through assignments to branch hospitals or medical alliances, and providing support for frontier medical work are important ways to improve professional identity.

Young medical professionals scored highly in the professional behavior dimension, with a total score of 16 (3) and an average of 4.26 (0.51). This dimension primarily investigates their self-assessment of professional capabilities, fulfillment of job responsibilities, and relationships with colleagues, reflecting their judgment of their professional skills and behaviors. This finding is consistent with Qu Shan’s research on professional identity and its influencing factors among doctors, where professional behavior had the highest average score among all dimensions of professional identity (27). The high score in professional behavior reflects the young medical professionals’ acknowledgment of the high technical and professional requirements of the healthcare industry. They recognize the need for continuous improvement in their professional skills and the fulfillment of job responsibilities through work practice.

Therefore, in emergency management situations, hospitals should not only handle task division and personnel scheduling but also provide necessary working conditions and humanistic care, focusing on the physical and psychological well-being of employees. Hospitals should offer solid support to frontline pandemic fighters. Measures include: (1) establishing temporary emergency working groups, such as temporary Party branches in makeshift hospitals, to comprehensively manage emergency pandemic responses; (2) implementing zoned and segmented management, with acting director overseeing daily operations, personnel management, pandemic prevention, and logistics, providing necessary living and working conditions and humanistic care to those in closed-loop management; (3) increasing pandemic allowances, issuing temporary work subsidies, and caring for dual-worker families by providing care packages for their families; and (4) offering material and spiritual rewards to employees who demonstrated dedication during the pandemic after its conclusion and giving preferential treatment in title evaluations and career development. By deploying these measures in a comprehensive, people-oriented manner, hospitals can enhance the humanistic care provided to young medical professionals, increase their professional identity, strengthen talent deployment, and promote sustainable hospital development. Focusing on young medical professionals provides a unique perspective on how early-career healthcare workers perceive their roles and identities when faced with unprecedented challenges. Understanding these influencing factors is essential for developing targeted interventions that can support this vulnerable group during crises. The global relevance of this study is substantial, as the findings can inform policies and support systems in similar contexts worldwide. By identifying the key factors that influence occupational identity, healthcare systems globally can implement strategies to enhance job satisfaction, reduce stress, and improve overall well-being among medical professionals during public health emergencies.

Importantly, this study is among the first to examine how perceived humanistic care and working condition satisfaction independently predict professional identity, beyond traditional demographic or workload factors. Our integration of these organizational-level predictors into a multidimensional identity model provides a novel framework for understanding resilience beyond individual-level coping strategies. The emphasis on structural and environmental support contributes to an evolving discourse on system-level interventions for workforce well-being.

Building on this framework, the study’s key findings highlight the substantial impact of public health emergencies on the occupational identity of medical youth. Although workload and direct exposure to COVID-19 patients were hypothesized to be stress-inducing based on prior literature, these factors did not show statistically significant associations with professional identity in our analysis. Nevertheless, their potential role in shaping occupational experiences remains theoretically important and may manifest under different contextual or temporal conditions. Similarly, perceived support from hospital administration—while not statistically significant in this dataset—remains conceptually relevant within the JD-R framework. Prior studies have emphasized the role of effective communication, resource availability, and mental health support in maintaining the well-being of healthcare workers during public health emergencies. These elements may influence professional identity indirectly or in combination with other unmeasured variables, and future longitudinal or qualitative studies could further clarify their roles.

Beyond organizational influences, personal attributes such as resilience and motivation also emerged as critical factors shaping professional identity. Resilient healthcare workers were better able to manage workplace stress and maintain a strong professional identity (38). Motivation, particularly internal motivation, was found to be essential for developing a robust occupational identity, with motivated individuals more likely to find personal and professional fulfillment in their roles. Despite these cumulative stressors, young medical professionals in this study maintained a strong sense of professional identity. Possible reasons for this include: (1) The hospital where the survey was conducted is a large general hospital with strong emergency response capabilities, providing a sense of professional security for young medical professionals; (2) During the public health emergency, the hospital’s infection management office and publicity department organized several online pandemic prevention training sessions and psychological counseling through platforms like WeChat and the internet, enhancing employees’ pandemic prevention skills; (3) The exemplary role of outstanding medical personnel who provided aid to Wuhan in 2020 inspired the spirit of professional dedication among young medical professionals in the context of pandemic response.

Moreover, this study found that satisfaction with the working conditions and humanistic care provided by the hospital is an important influencing factor for the professional identity of young medical professionals. Those who were satisfied with the hospital’s working conditions and humanistic care had a total professional identity score of 83, much higher than those who were dissatisfied. Under the impact of public health emergencies, the workload and pressure of young medical professionals increased significantly. Among them, 42% worked for more than 30 consecutive days, 69.30% felt that their work pressure had significantly increased, and nearly 60% experienced unit-based closed-loop management. The immense pressure of patient treatment and pandemic prevention, concerns about infection for themselves and their families, difficulties in returning home due to closed-loop management, and uncertainty about when the pandemic would end all caused significant physical fatigue and psychological anxiety for young medical professionals. This highlights the urgent need for organizational support and relief from their respective institutions.

However, the findings of this study have several implications for future research. First, there is a need for longitudinal studies to examine the long-term impact of public health emergencies on the occupational identity of medical professionals. Such studies can provide insights into how occupational identity changes over time and the factors that contribute to its development and maintenance. Second, future research should explore the effectiveness of different support systems and interventions in enhancing job satisfaction and reducing stress among healthcare workers. This can include examining the role of mental health programs, resource availability, and communication strategies in supporting healthcare workers during crises.

Additionally, future research should investigate the impact of organizational culture on occupational identity. Understanding how different organizational cultures influence the professional identity of healthcare workers can inform the development of targeted interventions to support their well-being. Finally, there is a need for research on the role of educational programs in shaping occupational identity. Examining how different educational curricula and training programs influence the professional identity of medical students and professionals can provide valuable insights for improving medical education. Although this study was conducted in a single megahospital in Shanghai, its findings resonate with health systems globally, particularly in countries managing resource limitations, workforce burnout, and operational disruptions during public health emergencies. By identifying modifiable organizational predictors of professional identity, such as perceived care and workplace conditions, we provide evidence to inform scalable strategies for retaining and supporting young healthcare professionals in similar high-stress contexts.

## Conclusion

This study provides a novel and thorough examination of the occupational identity of medical youth in large general hospitals in Shanghai during public health emergencies. By utilizing quantitative data, the research highlights the substantial impact of organizational factors such as satisfaction with humanistic care and working conditions on the occupational identity of young medical professionals. Further research is necessary to clarify the roles of workload, institutional support, resilience, and motivation, as these were either not statistically significant or not assessed in the current analysis. The insights gained from this study can inform targeted interventions and organizational strategies aimed at enhancing job satisfaction and overall well-being among healthcare workers during crisis periods.

Overall, this study makes a substantial contribution to the understanding of the occupational identity of medical youth during public health crises, offering valuable insights for healthcare systems worldwide. By addressing the identified limitations and building on the findings, future research can further clarify the complex interplay of factors influencing occupational identity and inform the development of effective support systems for healthcare professionals.

## Data Availability

The original contributions presented in the study are included in the article/supplementary material, further inquiries can be directed to the corresponding authors.
